# Effect of antibiotic pre-treatment and pathogen challenge on the intestinal microbiota in mice

**DOI:** 10.1186/s13099-016-0143-z

**Published:** 2016-11-18

**Authors:** Tadasu Iizumi, Takako Taniguchi, Wataru Yamazaki, Geraldine Vilmen, Alexander V. Alekseyenko, Zhan Gao, Guillermo I. Perez Perez, Martin J. Blaser

**Affiliations:** 1 Department of Medicine, New York University School of Medicine, New York, NY USA; 2 Department of Agriculture, University of Miyazaki, Miyazaki, Japan; 3 Universite Montpellier, Montpellier, France; 4 Department of Microbiology, New York University School of Medicine, New York, NY USA; 5 6026 W VAMC, 423 East 23th Street, New York, NY 10010 USA

**Keywords:** Microbiome, Antibiotic treatment, Pathogen challenge, Penicillin, Ciprofloxacin, *Campylobacter jejuni*, *Acinetobacter baumannii*, High-throughput sequencing, Next-generation sequencing

## Abstract

**Background:**

More than 50 years after the discovery of antibiotics, bacterial infections have decreased substantially; however, antibiotics also may have negative effects such as increasing susceptibility to pathogens. An intact microbiome is an important line of defense against pathogens. We sought to determine the effect of orally administered antibiotics both on susceptibility to pathogens and on impact to the microbiome. We studied *Campylobacter jejuni*, one of the most common causes of human diarrhea, and *Acinetobacter baumannii*, which causes wound infections. We examined the effects of antibiotic treatment on the susceptibility of mice to those pathogens as well as their influence on the mouse gut microbiome.

**Results:**

In C57/BL6 mice models, we explored the effects of pathogen challenge, and antibiotic treatment on the intestinal microbiota. Mice were treated with either ciprofloxacin, penicillin, or water (control) for a 5-day period followed by a 5-day washout period prior to oral challenge with *C. jejuni* or *A. baumannii* to assess antibiotic effects on colonization susceptibility. Mice were successfully colonized with *C. jejuni* more than 118 days, but only transiently with *A. baumannii*. These challenges did not lead to any major effects on the composition of the gut microbiota. Although antibiotic pre-treatment did not modify pathogen colonization, it affected richness and community structure of the gut microbiome. However, the antibiotic dysbiosis was significantly reduced by pathogen challenge.

**Conclusions:**

We conclude that despite gut microbiota disturbance, susceptibility to gut colonization by these pathogens was unchanged. The major gut microbiome disturbance produced by antibiotic treatment may be reduced by colonization with specific microbial taxa.

**Electronic supplementary material:**

The online version of this article (doi:10.1186/s13099-016-0143-z) contains supplementary material, which is available to authorized users.

## Background

The mammalian intestine hosts a complex and diverse microbial community [1, 2]. This ecosystem interacts extensively with its host, with substantial physiological and pathological effects [3]. For example, the gut microbiota is crucial to the host’s ability to resist colonization by pathogens [4, 5], although the mechanisms involved are incompletely characterized [6].

The clinical use of antibiotics has become massive in recent decades [7]. Their use increases susceptibility to acquired pathogen, although the underlying mechanisms are not well-understood [8]. Antibiotics change the composition of microbiota in the GI tract [9], affecting metabolic, hormonal, and immunological interactions between community and host, as well as intra-community interactions [10–12]. Separately, or together, these effects may increase host susceptibility to infection by introduced pathogens.

*Campylobacter jejuni* are Gram-negative, microaerophilic, curved rods, that commonly cause diarrheal illnesses, and can affect previously healthy hosts [13–15]. *Acinetobacter baumannii*, non-fermentative Gram-negative cocobacilli, have become increasingly common nosocomial pathogens, especially in intensive care units (ICUs) [16, 17]. The high prevalence of intestinal *A. baumannii* colonization in ICU patients suggests that the colon may be an important reservoir [18].

In this study, we developed mouse models involving colonization with these human pathogens to address three questions germane to colonization resistance; (1) what is the extent to which pathogens such as *C. jejuni* or *A. baumannii* colonize the GI tract of mice; (2) how does such colonization affect the gut microbiota; and (3) does pre-treatment with antibiotics change microbiota compositions and affect susceptibility to colonization by these pathogens?

## Methods

### Study design

Three related experiments were conducted in which control (untreated) mice were compared to experimental mice that were challenged by a pathogen, either alone, or in conjunction with antibiotic pre-treatment. In experiment #1, 7 week-old mice were challenged with either *A. baumannii* or *C. jejuni* (Additional file 1: Figure S1, panel A). In experiment #2, 6 week-old mice were challenged with one of three strains of *C. jejuni* that varied based on their mouse-passage histories (Additional file 1: Figure S1, panel B). In experiment #3, mice were exposed first to an antibiotic regime of either penicillin or ciprofloxacin or neither (control), and then were challenged with either *A. baumannii* or *C. jejuni,* or remained unchallenged (Additional file 1: Figure S2).

### Mice

Female C57BL/6NJ mice were obtained from Jackson Laboratories at ~5 to 6 weeks of age and allowed to adjust to the NYU animal facility for 1 week. The animals then were used in experiment #1 (Additional file 1: Figure S1, panel A). In addition, animals originally received from Jackson Laboratories were used for breeding at the NYU animal facility, and the offspring females were used for experiments #2 and 3 (Additional file 1: Figure S1, panel B). In experiment #3, 10 days prior to bacterial challenge, mice were given water containing penicillin VK (0.167 mg/ml; Sigma Aldrich, St Louis MO, USA), or ciprofloxacin (0.13 mg/ml; Acros Organics, Geel, Belgium), or no antibiotic (control) for 5 days. Water containers were changed twice during these 5 days to supply fresh antibiotics. The protocols for the mouse experiments included in this study were approved by the New York University School of Medicine Institutional Animal Care and Use Committee (IACUC).

### Bacterial strains used for mice inoculation

*Campylobacter jejuni* strain 81-176, that was originally isolated from a milkborne outbreak of human campylobacteriosis [19], and has been used in human volunteer studies [20], was used in all three experiments. For experiment #2, we used two additional *C. jejuni* strains that were recovered from mice experimentally inoculated with strain 81-176 in experiment #1. *Campylobacter jejuni* strain MP-10 was isolated from mouse stool 42 days after colonization, and *C. jejuni* strain Cecum J1 was isolated from the cecum of a mouse 119 days after colonization. All *C. jejuni* strains were cultured for 48 h on Skirrow agar (Becton–Dickinson, Franklin Lakes NJ, USA) under microaerobic conditions at 37 °C. Cultures then were resuspended in phosphate-buffered saline (PBS; pH 7.2) and adjusted to a concentration of 10^8^ CFU (by OD_600_) in 400 µl, which was introduced via oral gavage to test mice. Control mice received an oral gavage of 400 µl of PBS.

*Acinetobacter baumannii* strain 11-1, used in experiments #1 and 3, was a recent clinical isolate obtained from the New York University Langone Medical Center (NYULMC) Clinical Microbiology Laboratory. *Acinetobacter baumannii* was cultured for 24 h using Columbia sheep blood agar (BD) and CHROM agar Acinetobacter Base (DRG International, Springfield NJ, USA) under aerobic conditions at 37 °C. *Acinetobacter baumannii* was resuspended in PBS and adjusted to a concentration of 1.3 × 10^11^ CFU (by OD_600_) per 400 µl to create the oral gavage inoculum.

### Fecal specimen collection, culture and DNA extraction

In all experiments, fecal specimens were collected from mice before and after pathogen challenge (Additional file 1: Figures S1, S2), and were either immediately cultured or frozen at −20 °C. About 20 mg of feces were resuspended in 1 ml of PBS and vortex-mixed for 30 s at room temperature. From this stock suspension, tenfold dilutions were made in PBS. Aliquots (100 µl) of the 10^0^, 10^−2^, and 10^−3^ dilutions were plated on CHROM agar for *A. baumannii* and on Skirrow agar for *C. jejuni*. Plates were incubated under the conditions indicated above and colony counts were reported as CFU/mg stool. Fecal DNA also was extracted from a 20 mg aliquot of mouse feces using the PowerSoil DNA Isolation Kit (MOBIO, West Carlsbad CA, USA), according to the manufacturer’s protocol. The concentration of extracted DNA was determined by Nanodrop 1000 (Thermo Scientific, Watham MA, USA), and DNA was stored at −20 °C until used.

### Quantitative PCR

Sets of qPCR primers (Table 1) were used to quantitate bacterial populations, based on the universal bacterial 16S rRNA sequences [21], *C. jejuni luxS* [22], and *A. baumannii oxa51* [23]. qPCRs were performed using 3.5 mM MgCl_2_, 0.4 ng/µl bovine serum albumin, 0.2 mM of each deoxynucleoside triphosphate, 10 pmol of each primer, 0.625 U *Taq* DNA polymerase (Qiagen, Valencia CA, USA), and 2 µl extracted DNA in a final 20-µl volume of SYBR green master mix. qPCR conditions included 5 min at 94 °C and 45 cycles of 10 s at 94 °C, 10 s at 60 °C (*C. jejuni* and *A. baumannii*) or 56 °C (total bacteria). All assays were performed using a Light Cycler 480 (Roche Diagnostic Corporation, Indianapolis IN, USA). Bacterial numbers were determined using standard curves based on serial dilutions of cloned PCR products. Each sample was tested at least twice, and the results were analyzed using the Rotor-Gene 3000 v.6.1.81 software.

**Table 1:** Primers used for PCR in this study

Target	Primer designation	Primer sequence^a^
Total bacteria 16S rRNA	519F	GGACTACCVGGTATCTAAKCC
	785R	CAGCAGCCGCGGTRATA
*C. jejuni* *luxS*	F	AGCGATCAAAGCAAAATTCC
	R	GGCAATTTGTTTGGCTTCAT
*A. baumannii* *Oxa*-*51*	F	TTTAGCTCGTCGTATTGGACTTGA
	R	GCCTCTTGCTGAGGAGTAATTTTT

^a^V = A + C + G, K = G + T, R = A + G

### Library preparation for high-throughput sequencing (HTS)

All samples were amplified and barcoded for multiplex pyrosequencing using primers targeted to the V4 region of the bacterial 16S rRNA gene under uniform PCR conditions that included 3 min at 94 °C and 45 cycles of 45 s at 94 °C, 60 s at 50 °C, and 90 s at 72 °C with final extension for 10 min at 72 °C [24]. We used forward primer (AAT GAT ACG GCG ACC ACC GAG ATC TAC ACT ATG GTA ATT GTG TGC CAG CMG CCG CGG TAA) that includes a 5′ Illumina adaptor, forward primer pad, 2 bp linker and the 515F 16S rRNA primer, and reverse primer (CAA GCA GAA GAC GGC ATA CGA GAT NNNNNNNNNNNN—AGT CAG TCA G-CC-GGA CTA CHV GGG TWT CTA AT) that includes the Illumina 3′ adapter with 12-nt error-correcting Golay barcode, reverse primer pad, 2 bp linker and the 806R 16S rRNA primer. We ran PCR in triplicate using 0.2 µM of the primers, 1 µl of template and 1X HotMasterMix (5 PRIME, Gaithersburg MD, USA), and cleaned the products using a PCR Purification Kit (Qiagen) after pooling. Cleaned PCR products were quantified using the Qubit dsDNA HS Assay Kit (Invitrogen™, Eugene OR, USA), then adjusted to an optimal molarity as described. Sequencing was performed using the Illumina MiSeq platform in the NYULMC Genome Technology Core.

### Taxonomic and ecological analyses

We analyzed all sequence data using the QIIME software (version Mac Qiime 1.8.0) [25]. After filtering procedures, similar sequences were clustered into operational taxonomic units (OTUs) using an open reference approach with UCLUST [26] against the Greengenes Core set. A representative sequence was then aligned using PyNAST, and FastTree created phylogenetic trees. Rarefaction analysis used Chao-1 and whole PD to measure α-diversity. Unweighted UniFrac distances were calculated to assess β-diversity; the Unweighted paired group method with arithmetic mean (UPGMA) was performed for UniFrac-based jackknifed hierarchical clustering. Principal coordinates analysis (PCoA) of UniFrac distance matrices provided graphical representation using a KiNG, ANOVA was used to compare OTU and genus-level abundances, and Linear discriminant analysis (LDA) effect size (LEfSe), a tool that can compare differences of relative abundance between ≥2 biological conditions [27], also was used for analysis.

## Results

### Quantifying bacteria in fecal DNA

Assessing total DNA concentrations using Nanodrop and total bacterial log_10_ copy number/ng DNA by qPCR, we found that they were similar between the control animals and those treated with either after antibiotic or pathogen challenge (Additional file 1: Figure S3, panels a–j). Thus, neither the antibiotic treatments nor the pathogen challenge affected the overall population size of the intestinal microbiota.

### Assessment of mouse intestinal colonization after challenge

**Fig. 1:**
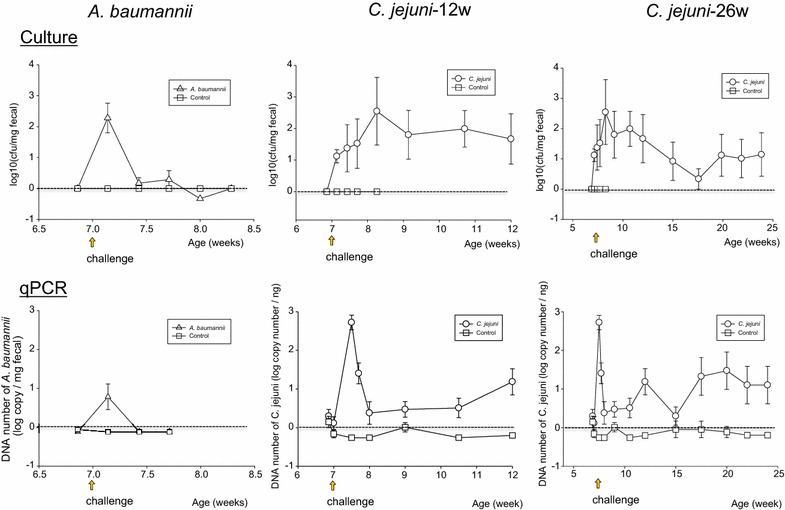
Quantitation of *A. baumannii* and *C. jejuni* intestinal colonization. *Upper panels* Detection by culture. *Lower panels* Detection by qPCR. Quantitation of *A. baumannii* is shown for weeks 7–8.5. Quantitation of *C. jejuni* is shown for weeks 7–12, and for weeks 7–26

Although we were able to colonize mice with *A. baumannii* (Fig. 1, left column), as evaluated by both culture and qPCR, colonization was transient and at low density. In contrast, we could achieve persistent mouse colonization with *C. jejuni* strain 81-176 (for ≥15 weeks), as confirmed by both culture and qPCR, until the experiment ended (Fig. 1, middle and right columns). Thus, the *A. baumannii* and *C. jejuni* strains used differed greatly in their ability to colonize the murine gut.

### Assessment of gut microbiome changes associated with *C. jejuni* challenge and cage effects

**Fig. 2:**
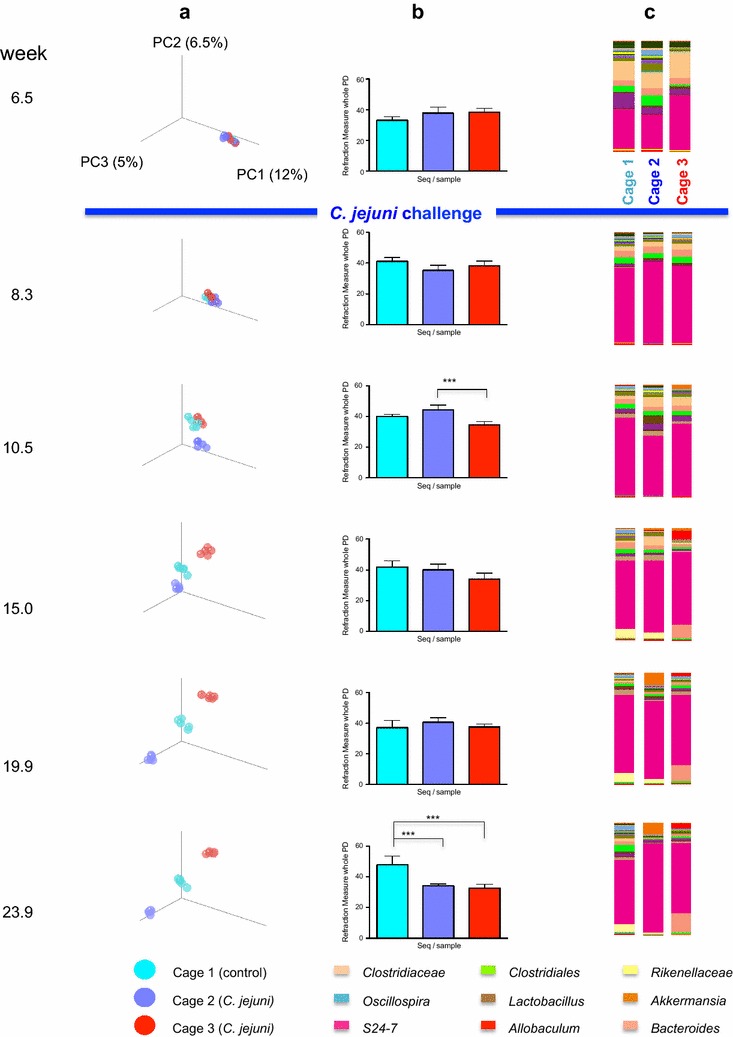
Assessment of change in gut microbiome following *C. jejuni* challenge. Mice were challenged with either PBS (cage 1), or *C. jejuni* (cages 2 and 3) at 7 weeks of age. Fecal pellets were collected serially, DNA extracted, and HTS performed on the Illumina Miseq platform. β-diversity (column A) α-diversity (column B). and relative taxon abundance at the genus level (color-coded, column C) are shown. *Light blue* indicates control group (cage 1); *dark blue* indicates *C. jejuni* group (cage 2); *red* indicates another *C. jejuni* group (cage 3)

We then assessed the gut microbiota in mice followed prospectively from 6.5 to 23.9 weeks (Fig. 2). In the initial pre-challenge samples, the bacterial communities were nearly identical in their community structure (column A). However, over time, the communities differentiated, based on the cage in which they were housed, and independently based on *C. jejuni* challenge or not. Next, assessing the species richness of the gut microbiota, all three cages were similar, although at the final time point, the *C. jejuni*-challenged group showed lower richness than controls (column B). All three groups were similar in relative abundance at the genus level (column C) except for late changes in *S24*-*7* abundance in cage 2.

### Quantitative differences in specific taxa

We next analyzed the HTS results using LEfSe, identifying specific taxa that showed significant differences between mice at the beginning (6.5 weeks of life) and end (23.9 weeks) of the experiment (Additional file 1: Figure S4). Multiple taxa within *Firmicutes, Bacilli,* and *Tenericutes* were significantly increased at the early time point in the three groups of mice with only cage-related minor variations. In contrast, taxa within *Bacteroides* and *Verrucomicrobia* were significantly higher at the later time point in all groups. Animals in cage 2 showed significantly increased *Proteobacteria*, consistent with our finding of persistent *C. jejuni* colonization. *Proteobacteria* were not increased in cage 1 (control) as expected, nor in cage 3 after *C. jejuni* colonization had spontaneously ceased.

### Detection of *C. jejuni* after mouse passage

Next, we investigated whether three *C. jejuni* strains with different passage histories varied in their abilities to colonize the mouse gastrointestinal tract after oral challenge (Additional file 1: Figure S5). All three *C. jejuni* strains showed similar kinetics; culture (panel A) and qPCR results (panel B) were consistent. Despite some variations in the kinetics of colonization, all three *C. jejuni* strains colonized the mouse gut to similar degrees.

### Effect of antibiotic treatment on mouse intestinal colonization

**Fig. 3:**
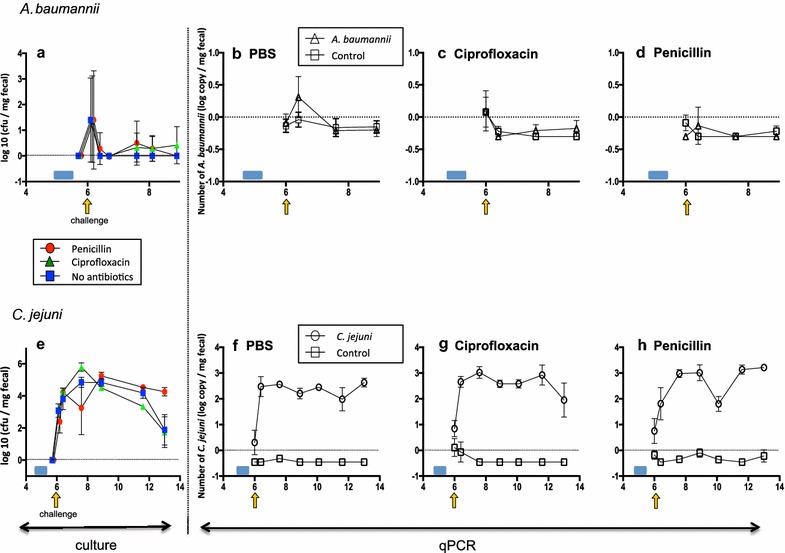
Quantitation of *A. baumannii* and *C. jejuni* in fecal pellets. **a**, **e** Culture detection. **b**–**d**, **f**–**h** qPCR assessment. Mice received 5 days of PBS (**b**, **f**), ciprofloxacin (**c**, **g**), or penicillin (**d**, **h**) and then were pathogen-challenged. The *blue box* indicates the period of antibiotic exposure. The *yellow arrow* indicates inoculation date of challenge with either *A. baumannii* (**a**–**d**) or *C. jejuni* (**e**–**h**)

We next studied the effect of pre-treatment of the mice with penicillin or ciprofloxacin, two antibiotics often used in clinical practice, on gut colonization with *C. jejuni* or *A. baumannii*. The antibiotics used to pre-treat the mice had no significant influence on intestinal colonization with either pathogen (Fig. 3). As in previous experiments, *A. baumannii* only transiently colonized the mice.

### Assessment of gut microbiota changes associated with bacterial challenge and antibiotic pre-treatment

**Fig. 4:**
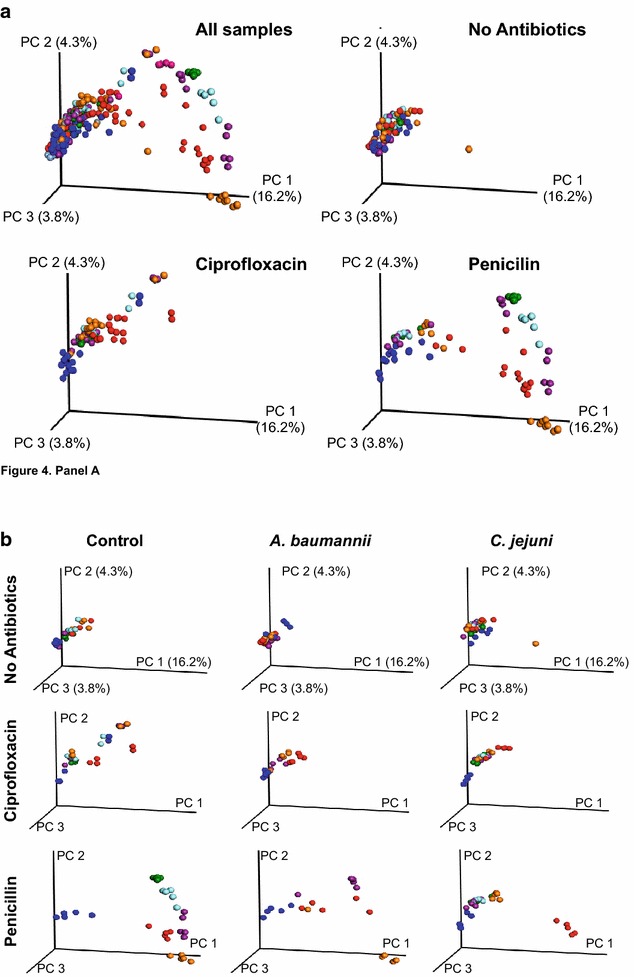
Assessment of antibiotic effects on intestinal microbial community structure (β-diversity). PCoA of the Unweighted UniFrac distances of microbial 16S rRNA sequences (V4 region) in fecal samples at six time points is shown. *Colored dots* indicate composites of the microbial community at 4.3 weeks (*blue*; before antibiotic treatment), 5.4 weeks (*red*; current antibiotic treatment), 6.1 weeks (*orange*), 7.6 weeks (*purple*), 10.1 weeks (*light blue*), or 13.0 weeks (*green*), respectively. **a** PCoA analysis sorted by antibiotic treatment, **b** *each column* indicates the antibiotic treatment group, and *each row* represents the bacterial challenge

We assessed the gut microbiome in mice followed for nearly 9 weeks. Assessment of β-diversity of the gut microbiota in the group of mice with no antibiotic treatment had nearly identical community structure at 6 different time points, indicating stable populations. However, mice treated with penicillin showed dramatic changes in β-diversity, across time. In contrast, mice treated with ciprofloxacin had minor effects in β-diversity (Fig. 4, panel A).

We next performed analysis of β-diversity of the gut microbiota in relation to antibiotic treatment and pathogen challenge. As before, mice with no antibiotic treatment had nearly identical community structure, regardless of pathogen challenge. In the group of mice receiving ciprofloxacin, the no challenge group had minor changes in β-diversity compared to either the *A. baumannii* or *C. jejuni* challenged groups. As above, mice treated with penicillin showed dramatic changes in community structure. However, mice treated with penicillin and challenged with *C. jejuni* changed in β-diversity and then recovered immediately. Similar but less dramatic effects were observed in mice treated with penicillin and challenged with *A. baumannii* (Fig. 4, panel B).

### Analysis of longitudinal inter-group phylogenic distances associated with bacterial challenge and antibiotic treatment

We observed minor β-diversity differences of the gut microbiota in the different mouse groups at time 1 (4.3 week) which was before antibiotic treatment and pathogen challenge. As such, we considered that the difference may be related to cage effects at that time point (Additional file 1: Figure S6, panel A). Next, we analyzed the intergroup variability at different time points in relation to antibiotic treatment and pathogen challenge, using the PERMANOVA metric. We did not observe significant differences in community structure in the mice challenged with either *A. baumannii* or *C. jejuni* without antibiotic pre-treatment (control groups) at any time point. In contrast, community structure in the mice treated with antibiotics and challenged with *A. baumannii* were significantly different from community structure before challenge. Furthermore, community structure of antibiotic-treated mice that were challenged with *C. jejuni* also were significantly different than pre-challenge (Data not shown). The community structure differences in the mice treated with penicillin and challenged with *C. jejuni* were significantly greater than before challenge (Additional file 1: Figure S7). Interestingly, these data suggest that the presence of *C. jejuni* minimized microbiome susceptibility to penicillin effects.

In mice without antibiotic pre-treatment, α-diversity was highest in the *C. jejuni*-challenged group; due to the short follow-up of the *A. baumannii* group, we could not assess changes. We consistently observed that in animals treated with penicillin, α-diversity was significantly increased in those challenged with *C. jejuni*; ciprofloxacin did not affect richness (Additional file 1: Figure S6, panel B).

**Fig. 5:**
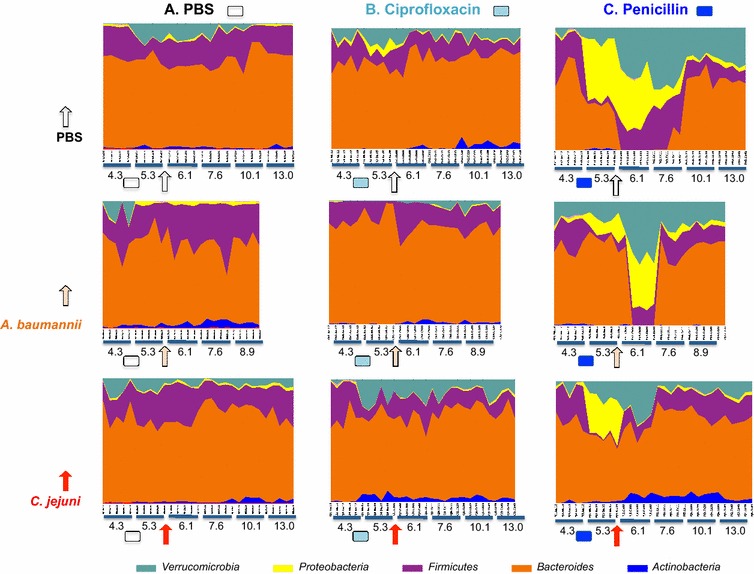
Relative abundance at phylum level of individual mice followed prospectively. *Each column* indicates treatment group and the *boxes* indicate time points of antibiotic treatment or not (PBS control). *Each row* represents the bacterial challenge and the *arrows* indicate time of challenge. The study period was up to 13 weeks for the animals challenged with *C. jejuni* and 9 weeks for the mice challenged with *A. baumannii*

Finally, the relative abundance of taxa in the control mice (not pre-treated with antibiotics), that were challenged with *A. baumannii* or *C. jejuni* (Fig. 5, column A) differed little from the (control) mice without challenge. There was a major effect on taxa abundances in mice pre-treated with penicillin, but this was significantly reduced after *A. baumannii*, or *C. jejuni* challenge (column C). In contrast, ciprofloxacin had much smaller effects (Fig. 5, column B).

## Discussion

In these studies, persistent colonization was achieved with *C. jejuni*, an intestinal pathogen, but not with *A. baumannii,* consistent with prior reports [28, 29]. We assessed the health status of the mice challenged with the pathogens and with or without antibiotic treatment and we did not notice any significant change in behavior or signs of disturbances in the health of mice that confirmed previous information that mice do not express disease after colonization with *C. jejuni* [30, 31].

Longitudinal assessment of bacterial gut community structure indicates that at early time points, essentially all mice were identical at the phylum level. However, as the experiments progressed, community structures varied, due to cage effects, and independently in relation to antibiotic treatment, or *C. jejuni* challenge.

However, the community richness in the gut microbiome was relatively constant, unaffected by cage, or antibiotic effects. Nevertheless, *C. jejuni* challenge increased richness, which was affected by penicillin pre-treatment. Mouse-adapted strains of *C. jejuni* were not better than a parental strain in mouse gut colonization. One hypothesis for the mechanism for the increased richness is that *C. jejuni* competes with dominant strains, permitting the more minor taxa to bloom. An alternative hypothesis is that *C. jejuni* infection stimulates production of anti-microbial peptides, as has been reported [32], and these select against the dominant taxa.

Antibiotic pre-treatment showed no effect on the capabilities of *C. jejuni* and *A. baumannii* to colonize the mice, which for *A. baumannii* was only transient. We recognized that variation in antibiotic concentration may cause different effects. However, in this study we used antibiotics equivalent of therapeutic doses used in humans to reach maximum antibiotic concentration in mice. Stable community structure was observed in mice for nearly 10 weeks whether challenged or not. Ciprofloxacin induced minor community-wide disturbances but penicillin induced major disturbances (Fig. 5).

In the absence of antibiotic pre-treatment, pathogen introduction did not affect relative abundances of the colonizing taxa either at the phyla (Fig. 5) or genus level (Data not shown). In contrast, antibiotic pre-treatment with ciprofloxacin produced minor disturbance of the gut microbiota when compare to penicillin. One explanation may be the lower activity of ciprofloxacin against anaerobes [33], in contrast to the known anti anaerobic activity of penicillin [34, 35]. Surprisingly, the introduction of *A. baumannii* or *C. jejuni* ameliorated the disturbances in the community structure, and facilitated the recovery to normality. We speculate that pathogen introduction affected either host responses that led to stereotypic changes, or to altered competition dynamics in the gut, favoring the status quo ante.

In conclusion, the susceptibility to gut colonization by *A. baumannii* and *C. jejuni* did not change despite the disruption of the gut microbiota of mice treated with antibiotics. The distruption of the gut mictobiota may be reduced by *C. jejuni* colonization but the mechanism is still unknown.

## Additional file

**Additional file 1.** Additional figures.

## Abbreviations

HTS: high-throughput sequencing
rRNA: ribosomal ribonucleic acid
OTUs: operational taxonomic units
PCoA: principal coordinates analysis
LEfSE: linear discriminant analysis (LAD) effect size
