# Supplementary material for: Effect of antibiotic pre-treatment and pathogen challenge on the intestinal microbiota in mice
Source: Gut Pathog. 2016 Nov 18;8:60. doi: 10.1186/s13099-016-0143-z (PMC5116224; doi:10.1186/s13099-016-0143-z)
Supplement: Supplementary file 1 — Additional file 1. Additional figures. [file 13099_2016_143_MOESM1_ESM.pdf]

Supplemental Figures

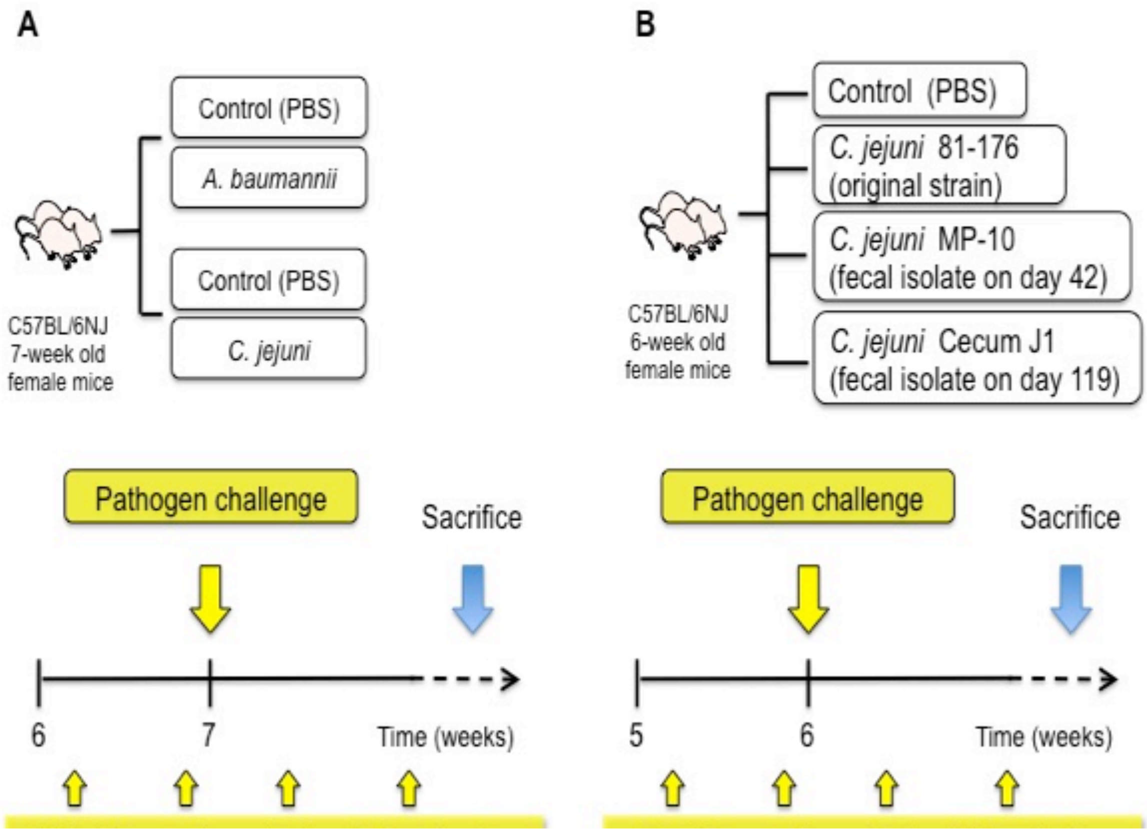

Figure S1. Experimental design for assessment of intestinal colonization of mice.

Panel A: C57BL/6 NJ mice were orally inoculated at 7 weeks of age with *A. baumannii* or *C. jejuni*. **Panel B:** C57BL/6 NJ mice were orally inoculated at 6 weeks of age with one of three *C. jejuni* strains that differed in passage history.

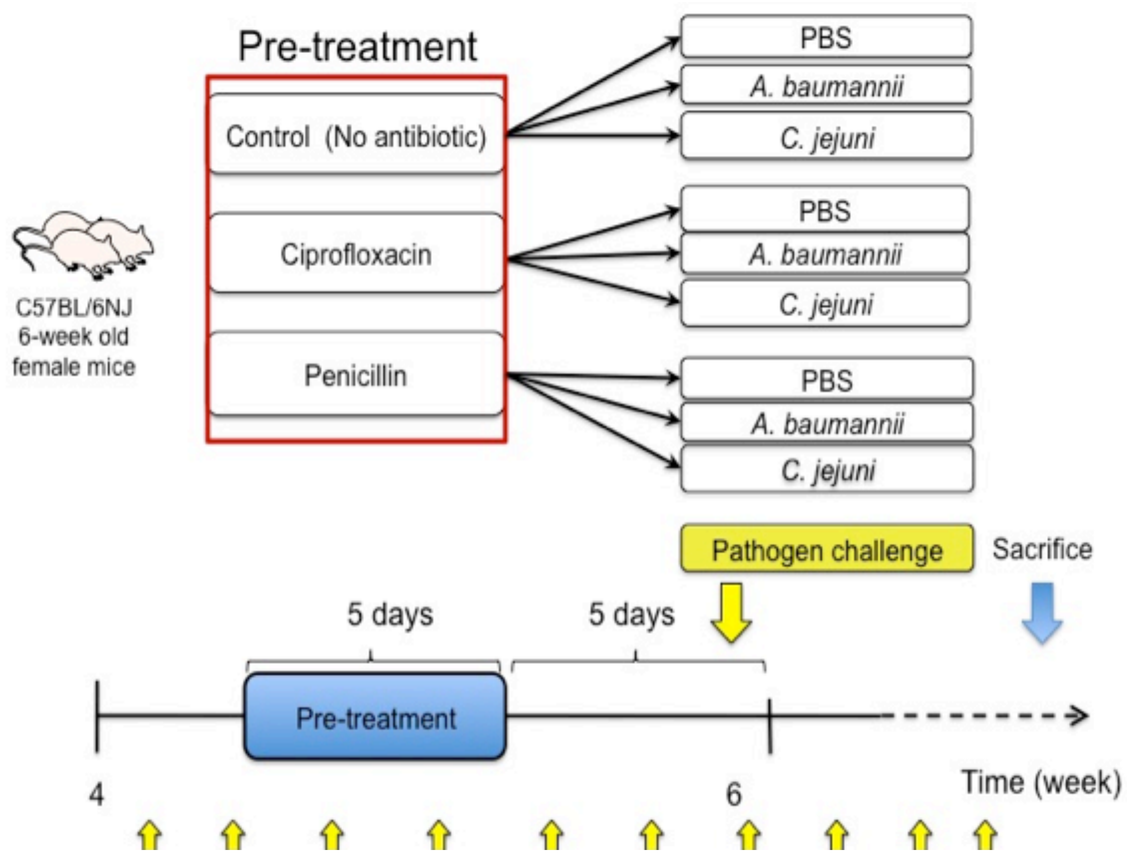

Figure S2. Experimental design to determine the effect of prior antibiotic treatment on mouse colonization by *A. baumannii* or *C. jejuni*.

Mice were given water containing penicillin VK, ciprofloxacin, or PBS for a 5 day period. Five days after the end of the antibiotic (or control) treatment, mice were inoculated with *A. baumannii*, *C. jejuni*, or with PBS as a control.

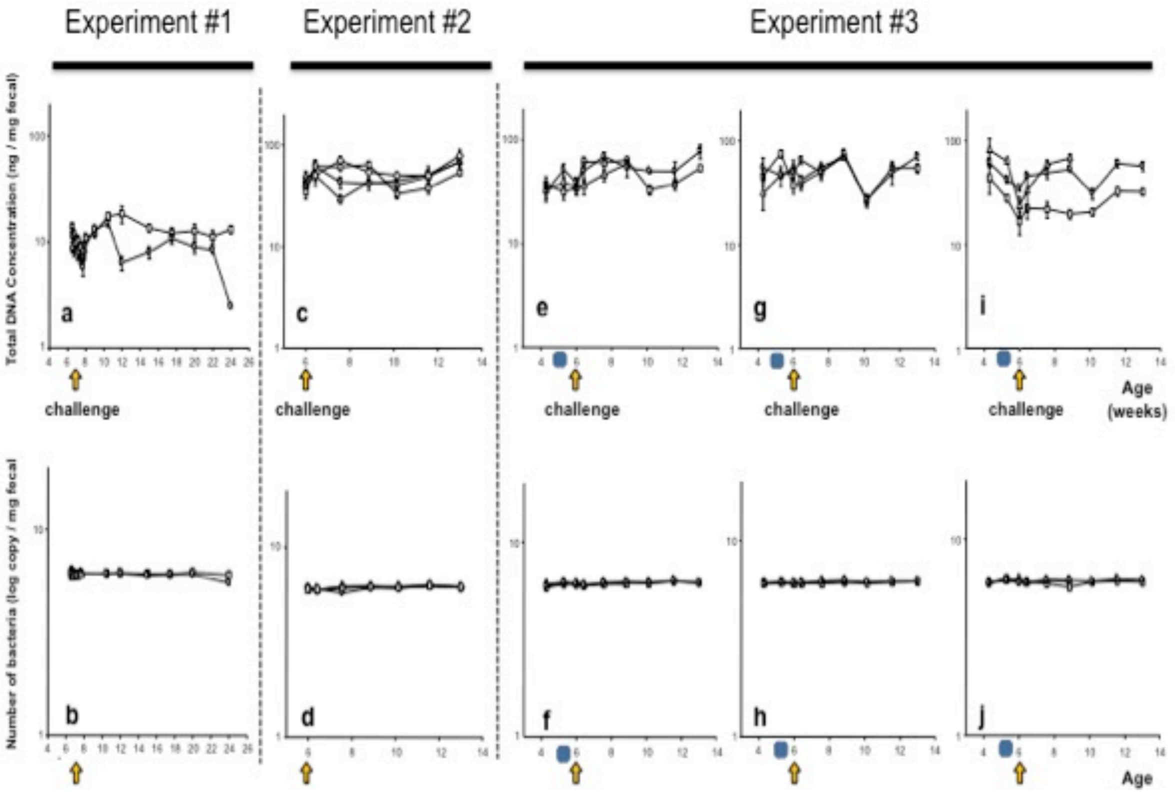

Figure S3. Quantitation of total bacteria in fecal pellets.

Top panels. Bacterial DNA concentration, determined by nanodrop. **Bottom panels**. Detection of total number of bacteria by qPCR. **Panels a and b**. Experiment #1. Mice received from Jackson Laboratories were inoculated with PBS (control), *C. jejuni* or *A. baumannii* at 7 weeks of age, with continuous follow-up until 24 weeks. **Panels c and d**. Experiment #2. Mice from NYU were inoculated with PBS (control) or with any of three strains of *C.jejuni* at 6 weeks of age, with continuous observation until 13 weeks. **Panels e to j**. Experiment #3. Challenge was performed in groups of 5 mice after receiving five days of either

PBS (**Panels e and f**), ciprofloxacin (**Panels g and h**), or penicillin (**Panels i and j**). The blue boxes indicate the period of exposure to antibiotics. Animals were challenged with *C. jejuni*, *A. baumannii,* or PBS (control). The arrows indicate the time of challenge.

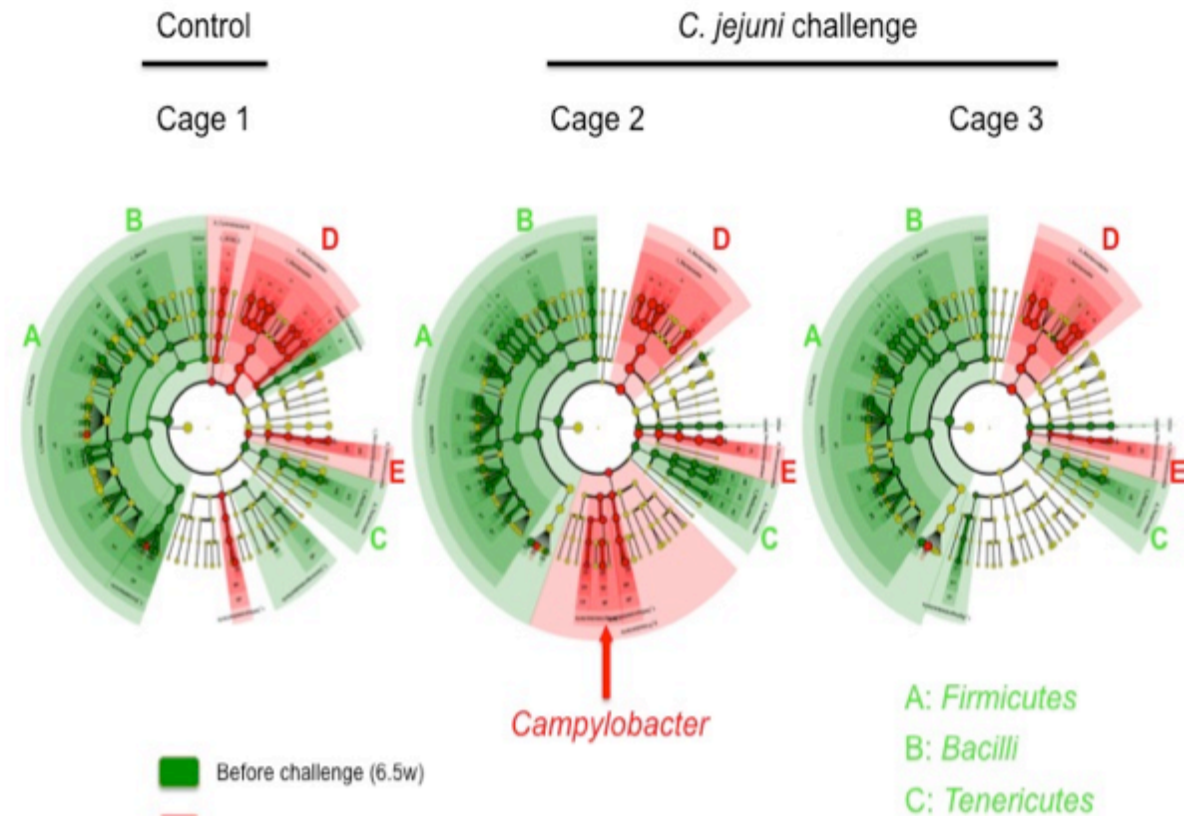

Figure S4. Comparative analysis of relative abundance of specific taxa using LEfSe. Relative abundance differences for mice in each cage at two time points: before challenge (6.5 weeks of age) and ~16 weeks after challenge (23.9 weeks of age). The cladograms show taxonomic representation of statistically significant differences over time, by cage. Each cladogram represents a cage as described in Figure 2. Colors: green represents bacterial taxa that were significantly more abundant before *C. jejuni* challenge; red represents taxa significantly increased at the end of the experiment, after the challenge.

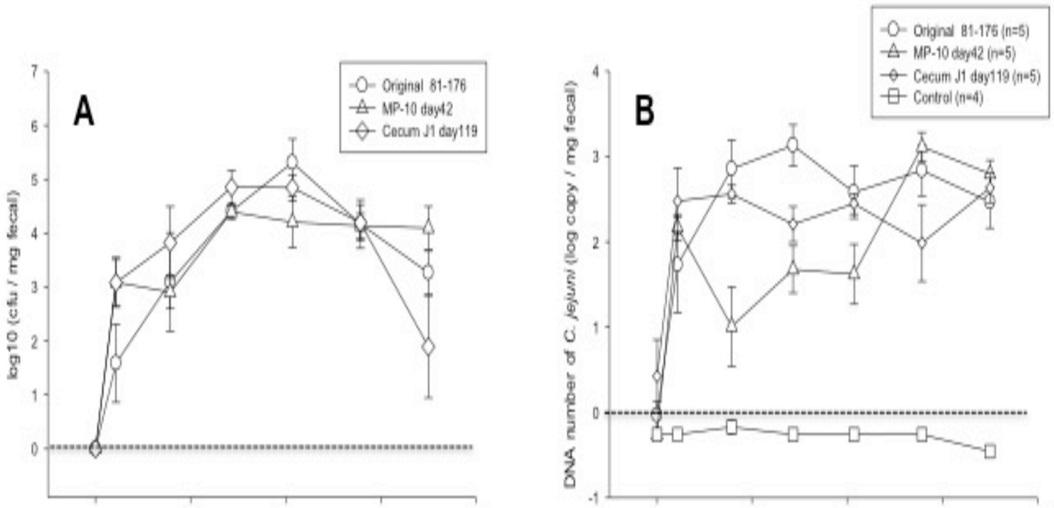

Figure S5. Assessment of *C. jejuni* intestinal colonization after challenge. To investigate colonization by mouse-passaged *C. jejuni* strains, mice were inoculated at 6 weeks of age with one of three different strains of *C. jejuni* or not (control). The arrow indicates time of challenge. Results of culture detection (**Panel A**) and qPCR (**Panel B**), are shown.

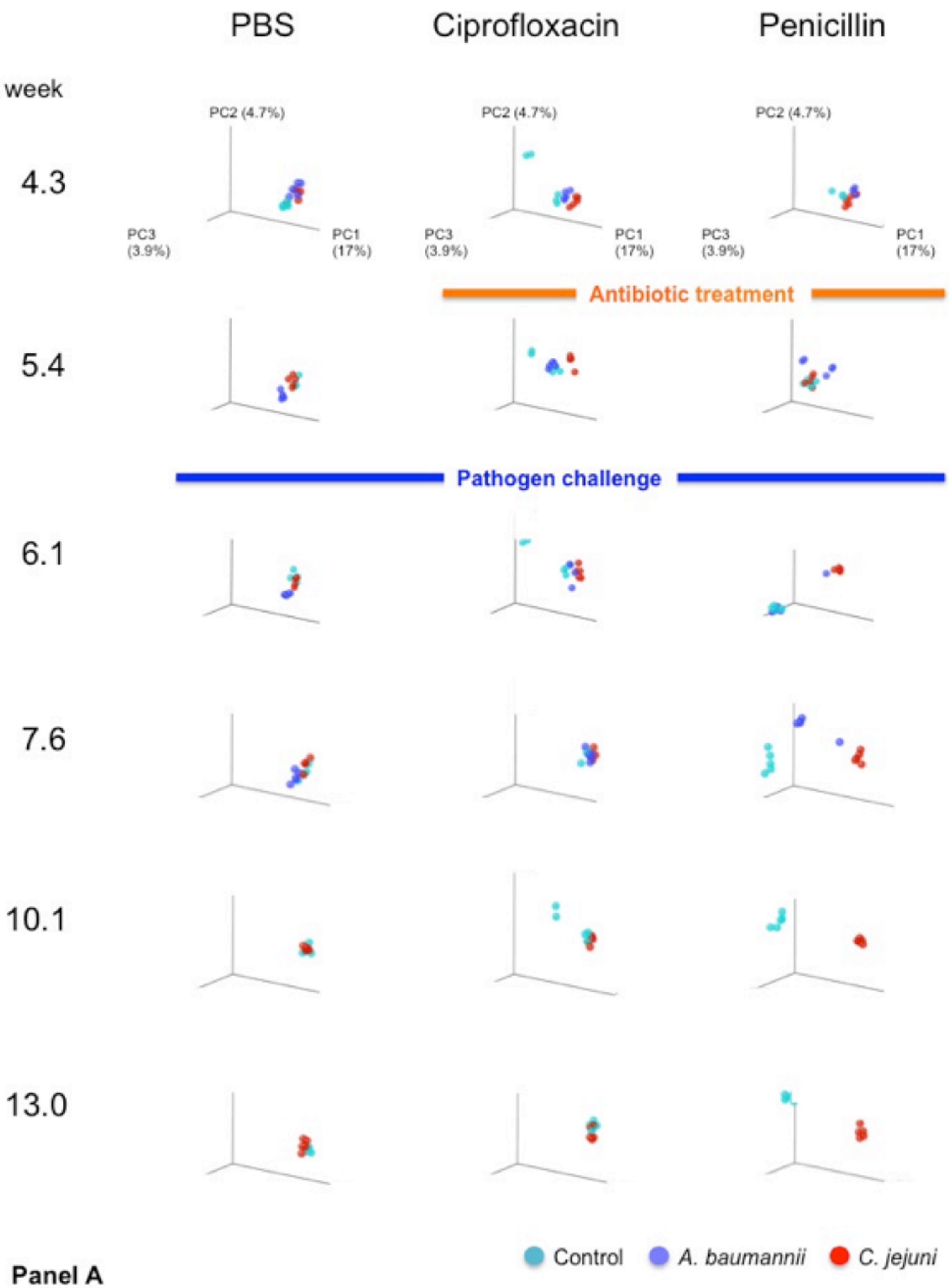
PBS
Ciprofloxacin
Penicillin
week
4.3
5.4
6.1
7.6
10.1
13.0
PC2 (4.7%)
PC3 (3.9%)
PC1 (17%)
Antibiotic treatment
Pathogen challenge
Control
A. baumannii
C. jejuni

Panel A

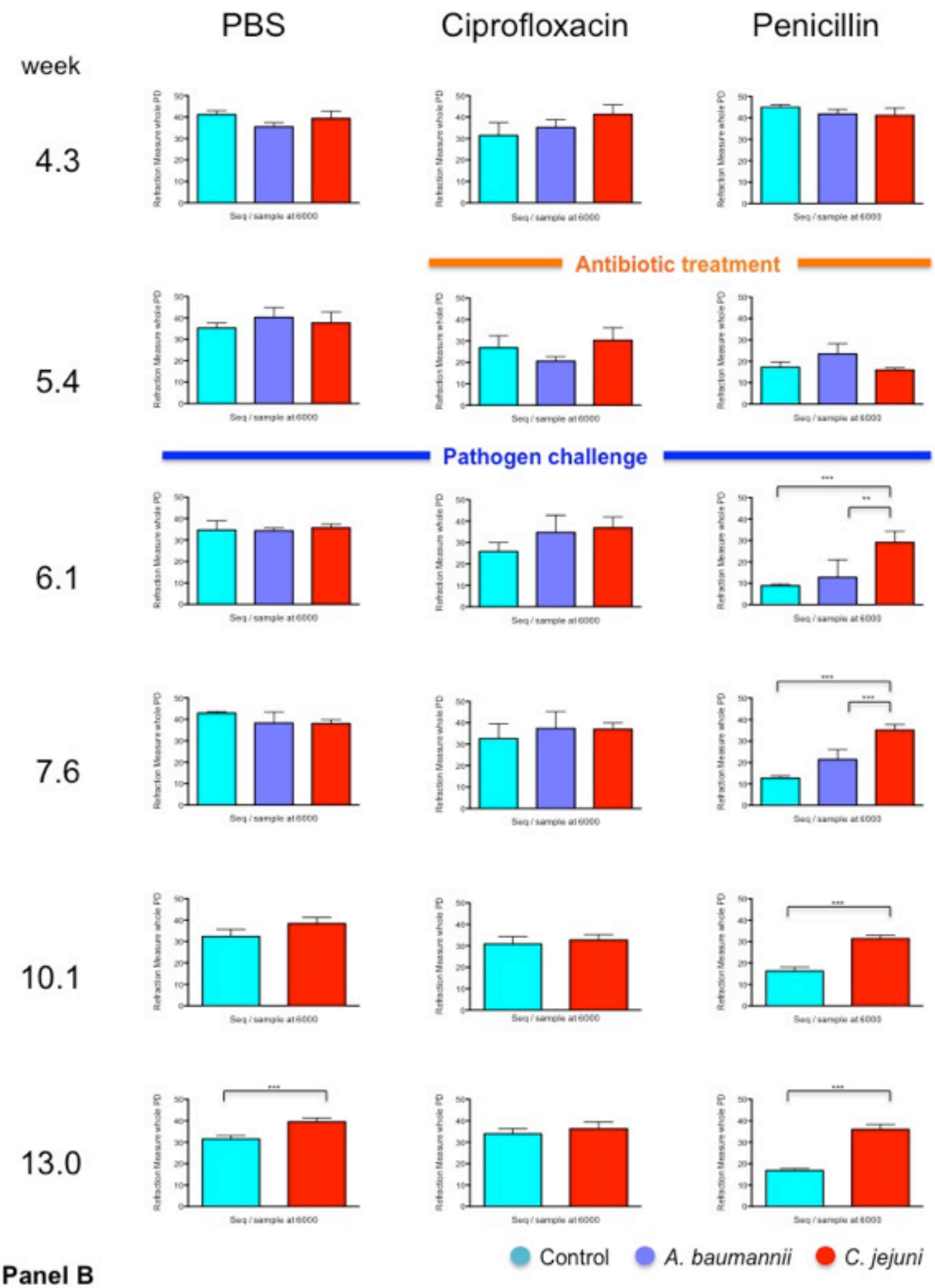

PBS
Ciprofloxacin
Penicillin
week
4.3
5.4
6.1
7.6
10.1
13.0
Antibiotic treatment
Pathogen challenge
Refraction Measure whole PD
Seq / sample at 6000
Control
A. baumannii
C. jejuni

Panel B

Figure S6. Assessment of longitudinal changes in microbial diversity associated with antibiotic treatment and bacterial challenge. Each row shows a different time point and each column indicates the treatment groups. The pathogen challenges are color-coded. Mice challenged with *A. baumannii* was studied only up to 7.6 weeks of age. **Panel A**: Beta-diversity. PCoA of the unweighted UniFrac distance of microbial 16S rRNA sequence (V4 region) in fecal samples is presented longitudinally from 4.3 to 13 weeks of age (Statistical analysis in **Supplement Figure 7**.). **Panel B:** alpha-diversity by whole PD metric associated with antibiotic treatment and bacterial challenge. Alpha-diversity was calculated using the whole PD evenness metric. * $p<0.05$, ** $p<0.01$, ***$p<0.001$, by ANOVA.

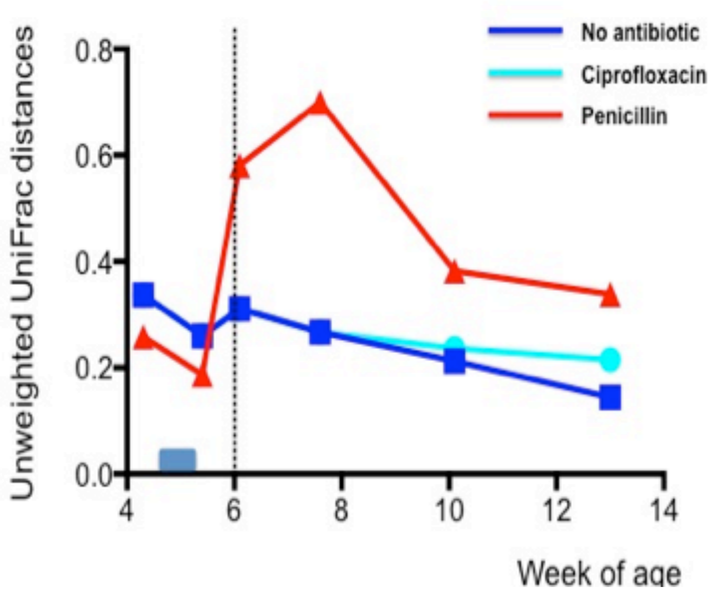

Figure S7. Comparison of the inter-group phylogenic distances over time. This figure indicates longitudinal changes in inter-group distances in beta diversity in mouse gut microbiota associated with *C. jejuni* challenge and antibiotic treatment. Unweighted Unifrac analysis of inter-group distance between mice challenged or not with *C. jejuni*, and treated or not with antibiotics is shown. The light blue line represents no antibiotics, dark blue is ciprofloxacin, and red is penicillin.
